# Host transmission dynamics of first- and third-stage *Angiostrongylus cantonensis* larvae in *Bullastra lessoni*

**DOI:** 10.1017/S0031182022000488

**Published:** 2022-07

**Authors:** Tsung Yu Pai, Wieland Meyer, Fraser R. Torpy, Shannon L. Donahoe, John Ellis, Richard Malik, Rogan Lee

**Affiliations:** 1 School of Life Sciences, University of Technology Sydney, Ultimo, NSW 2007, Australia; 2 Parasitology Laboratory, Centre for Infectious Diseases and Microbiology Lab Services, Level 3 ICPMR, Westmead Hospital, Westmead, NSW 2145, Australia; 3 Molecular Mycology Research Laboratory, Sydney Infectious Diseases Institute and Westmead Clinical School, Faculty of Medicine and Health, University of Sydney, Sydney, Australia; 4 Westmead Institute for Medical Research and Research and Education Network, Western Sydney Local Health District, Westmead, NSW 2145, Australia; 5 Curtin Medical School, Curtin University, Perth, Bentley, WA 6102, Australia; 6 Sydney School of Veterinary Science, Faculty of Science, University of Sydney, Sydney, NSW 2006, Australia; 7 Centre for Veterinary Education, B22, University of Sydney, Sydney, NSW 2006, Australia

**Keywords:** Angiostrongyliasis, *Angiostrongylus cantonensis*, *Bullastra lessoni*, infectivity, larval distribution, larval migration, viability

## Abstract

Given the importance of angiostrongyliasis as an emerging infectious disease of humans, companion animals, and wildlife, the current study focused on the transmission dynamics of first- and third-stage larvae of the parasitic nematode, *Angiostrongylus cantonensis*. The migration of infective larvae and their subsequent distribution within the Lymnaeidae snail, *Bullastra lessoni*, were investigated over time using microscopic examination of histological sections and fresh tissue. Snails were divided into four anatomical regions: (i) anterior and (ii) posterior cephalopedal masses, (iii) mantle skirt and (iv) visceral mass. The viability of free-swimming third-stage larvae, after their release from snail tissues, was evaluated *in vitro* by propidium iodide staining and infectivity by *in vivo* infection of Wistar rats. Snails were sequentially dissected over time to assess the number and anatomical distribution of larvae within each snail and hence infer their migration pathway. Herein, ongoing larval migratory activity was detected over 28 days post-infection. A comparison of infection rates and the larval distribution within the four designated snail regions demonstrated a significant relationship between anatomical region and density of infective larvae, with larvae mostly distributed in the anterior cephalopedal mass (43.6 ± 10.8%) and the mantle skirt (33.0 ± 8.8%). Propidium iodide staining showed that free-swimming third-stage larvae retained viability for between 4 and 8 weeks when stored under laboratory conditions. In contrast to viability, larval infectivity in rats remained for up to 2 weeks only. Knowledge gained from the current work could provide information on the development of new approaches to controlling the transmission of this parasite.

## Graphical abstract

**Figure:**
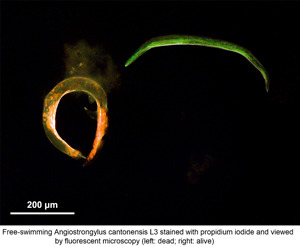

## Introduction

*Angiostrongylus cantonensis*, the rat lungworm, is a parasitic nematode, causing the disease angiostrongyliasis. In humans, this disease occurs following ingestion of raw or undercooked snails, paratenic hosts, or unwashed contaminated vegetables (Alicata and Brown, 1962; Heyneman and Lim, 1967; Rosen *et al*., 1967; Cowie, 2013*b*). This zoonosis manifests as eosinophilic meningitis and/or encephalitis (Alicata, 1988, 1991; Pien and Pien, 1999; Prociv *et al*., 2000; Ramirez-Avila *et al*., 2009; Thiengo *et al*., 2013; Cowie, 2013*b*; Defo *et al*., 2018; Prociv and Turner, 2018) and less commonly, ocular angiostrongyliasis (Sinawat *et al*., 2019). In severe cases, which occur most commonly in human infants and following the deliberate ingestion of live slugs, it can be either fatal (Morton *et al*., 2013; Prociv and Turner, 2018) or result in long-term neurological disability (Kwon *et al*., 2013; Epelboin *et al*., 2016). Human clinical cases have accumulated to over 2877 recorded infections worldwide by 2012 (Barratt *et al*., 2016). It has been suggested that this infection be added to the World Health Organisation's list of emerging infectious diseases and as a neglected tropical disease (Hotez *et al*., 2020). Additionally, more public health education is required to alert those at risk of being infected (Barratt *et al*., 2016; Johnston *et al*., 2019; Howe *et al*., 2021).

Various rat species are the definitive hosts for *A. cantonensis* (Mackerras and Sandars, 1955; Alicata and McCarthy, 1964; Wallace and Rosen, 1965). There are a broad range of slugs and terrestrial and freshwater snail species, particularly *Pomacea canaliculata*, *Parmarion martensi*, and *Achatina fulica* (Alicata, 1969, 1988; Wallace and Rosen, 1969*a*; Kliks and Palumbo, 1992; Thiengo *et al*., 2013; Cowie, 2013*a*), that can act as intermediate hosts for this parasite. Although the species, *Angiostrongylus mackerrasae*, was incorrectly identified as *A. cantonensis*, their life cycles in rats are similar, as originally described in Mackerras and Sandars (1955), and confirmed by subsequent studies (Jindrák, 1968; Wallace and Rosen, 1969*b*; Bhaibulaya, 1975). Subsequently, a broad range of other vertebrate hosts, including humans, have been shown to become infected by ingesting infected gastropods (Gardiner *et al*., 1990; Kim *et al*., 2002; Ma *et al*., 2013; Cowie, 2013*b*; Spratt, 2015; Walker *et al*., 2015; Wun *et al*., 2021*b*).

To contain transmission of angiostrongyliasis between snail and vertebrate hosts, a more comprehensive understanding of the actual mode of transmission to the intermediate snail host and the vertebrate host is needed. First and foremost, *Angiostrongylus costaricensis* first-stage larvae (L1), a species closely related to *A. cantonensis*, can infect the snail by entering from the mouth and penetrating the digestive tract or by directly penetrating the tegument and migrate in the snail body (Thiengo, 1996; Mendonça *et al*., 1999; Montresor *et al*., 2008). Although several studies have shown the distribution of *A. cantonensis* larvae in snails (Yousif *et al*., 1980; Tesana *et al*., 2008; Jarvi *et al*., 2012; Chan *et al*., 2015), the mechanism of entry and migration within the snail intermediate host has not been confirmed. Secondly, an earlier study has conjectured that rainwater and drinking water could be a source of transmission of larvae to humans (Alicata and Brown, 1962). In subsequent years, three further studies demonstrated the infection of rats by free-swimming, viable third-stage larvae (L3) 2 days after their release from snail fragments into freshwater. These larvae were shown to survive for at least 7 days (Richards and Merritt, 1967), and larvae exuded from the terrestrial *A. fulica* submerged in water for 60 hours remained infective (Crook *et al*., 1971). Infectivity of L3 in freshwater was subsequently supported by similar findings from two other lungworm species (*Aelurostrongylus abstrusus* and *Troglostrongylus brevior*) (Giannelli *et al*., 2015). This route of transmission was considered to be important in Hawaii, as one naturally infected terrestrial semi-slug (*P. martensi*) could potentially shed more than 300 L3 after 5 days immersion in rainwater (Howe *et al*., 2019). Furthermore, it is well known that gastropods often get washed into rainwater storage tanks where they drown. It is not yet known how long free-swimming L3 remain alive and infective.

The primary aim of this study was to understand the L1 and L3 transmission dynamics of *A. cantonensis*. We sought to investigate the mode of entry of L1 into a freshwater snail, how larvae are distributed within the snail, and the viability and infectivity of free-swimming L3 in freshwater.

## Material and methods

### Angiostrongylus cantonensis

The *A. cantonensis* isolate used in this study originated from a wild rat (*Rattus norvegicus*) caught near the Taronga Park Zoological Gardens in Sydney 30 years ago (cited in Červená *et al*., 2019). The mitochondrial genome of this isolate (SYD.1) was reported in the aforementioned study. The life cycle was maintained through laboratory-reared snails and rats, using the processes discussed below.

### Snails

*Bullastra lessoni* (family: Lymnaeidae), previously placed in the genus *Austropeplea*, is a gastropod native to Australia. This species was thought to consist of two morphologically and phylogenetically distinct lineages, divided between eastern and northern Australian populations (Puslednik *et al*., 2009), but these are now considered to be distinct species with the northern one being *Bullastra vinosa* (see Ponder *et al*., 2020). *Bullastra lessoni* was originally collected from a backyard pond in Wyong, NSW (33°17′S, 151°26′E). Snails were bred in the laboratory and isolated from any rodent contact. All snails were maintained at 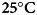 and 70 − 80% humidity in an aquarium tank (located in the Animal House, Westmead Hospital, Sydney, Australia), equipped with an air pump and a layer of crushed marble sediment. Washed lettuce was provided as a food source *ad libitum*. The tank was routinely rinsed with distilled water to remove juvenile snails, snail eggs, and lettuce residues.

### Infection of snails for larval migration and distribution experiments

*Angiostrongylus cantonensis* L1 were harvested from infected rat faeces by the Baermann technique (Mackerras and Sandars, 1955; Barçante *et al*., 2003) and identified by light microscopy of wet faecal preparations. Larvae were washed twice using reverse-osmosis (RO) water. A total of 160 snails, with an average weight of 0.37 g (*n* = 12 snails; median = 0.36 g; range = 0.29 − 0.49), were placed in a single covered Petri dish (18.5 cm in diameter) and exposed to 40 000 L1 contained in 100 mL of RO water for 4 hours. The Petri dish was intermittently agitated, to encourage equal exposure of all snails to larvae. Snails were then washed in RO water to remove free larvae on their surface, and the snails were maintained in a separate aquarium tank as the source of the larval migration and distribution experiments.

### Larval migration

**Fig. 1.**
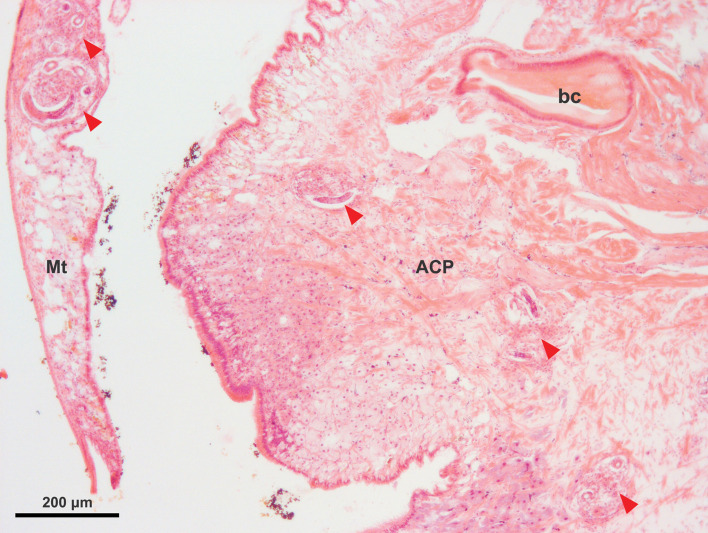
H&E-stained section of *Bullastra lessoni* showing *Angiostrongylus cantonensis* L1 (5 days post-infection). Larvae are marked with red arrows. The mantle skirt (Mt), anterior cephalopedal mass (ACP) and buccal cavity (bc) are shown.

Infected snails (*n* = 96) were collected in groups of 4 and fixed in 20 mL of 10% neutral-buffered formalin at successive increasing time intervals up to 28 days (collection times = 0, 0.5, 1, 2, 3, 4, 20, 23, 28, 43, 51, 67, 75 hours; 4, 5, 6, 7, 8, 9, 10, 13, 17, 22, 28 days post-infection). The underlying soft tissues of *B. lessoni* were carefully extricated without visible damage. Blunt forceps were used to puncture and fracture the shell. Fragments were lifted gently away from the snail organs, similar to the process employed by Lőw *et al*. (2016). This allowed for better fixation of the snail tissue. Formalin-fixed snails were processed for sectioning by standard histological methods. Six consecutive sagittal sections around the midline were mounted on glass slides, stained with haematoxylin and eosin (H&E), and examined using light microscopy (Fig. 1).

### Larval distribution

**Fig. 2.**
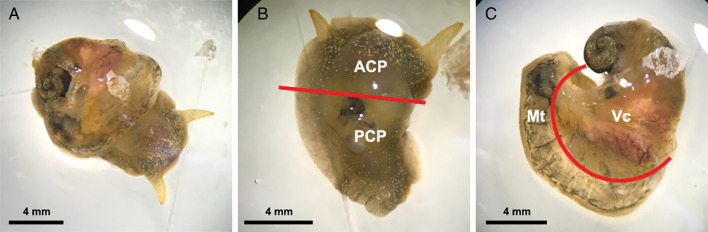
Examples of *Bullastra lessoni* snail dissection. (A) Whole snail after removal of the shell. (B) Anterior (ACP) and posterior (PCP) cephalopedal mass. (C) Mantle skirt (Mt) and visceral mass (Vc). Red lines represent the cuts made to divide the snail into four regions.

**Fig. 3.**
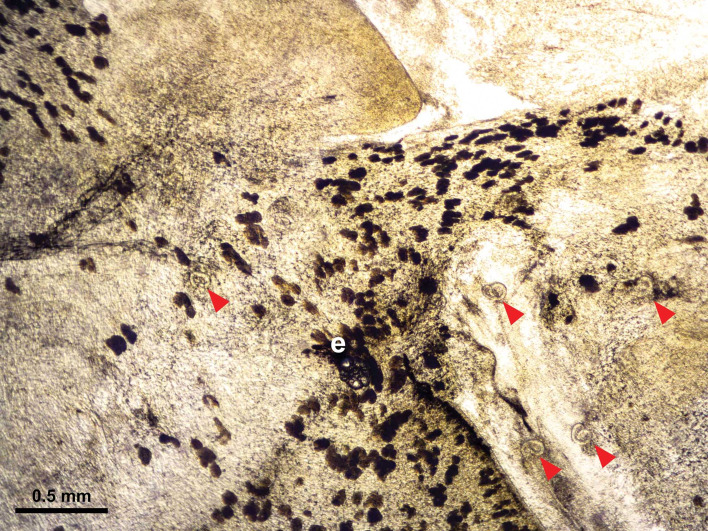
Light microscopic image of *Angiostrongylus cantonensis* L3 in *Bullastra lessoni* snail tissue. Five larvae, marked in arrows, are embedded in the fresh tissue. A part of the anterior cephalopedal region of snail is shown, and the eye (e) of the snail is situated lower to the centre of the figure.

For this experiment, 15 infected snails were collected from the aquarium at least 5 weeks post-infection, at which time larvae in the snails had moulted twice and developed into L3 (Lv *et al*., 2009; Thiengo *et al*., 2013). The systemic anatomy of the family Lymnaeidae as described in Ponder and Waterhouse (1997) is used here. The general anatomical features of *B. lessoni* are similar to *Lymnaea catascopium* (a confamilial species) (Fig. 2 in Walter, 1969), except that in *B. lessoni* the transverse band is noticeably much more ventral. Initially, the shell of the freshly collected snail was removed (Fig. 2A), followed by snail dissection. The first cut was made right below the transverse band, the tissue connecting the cephalopedal mass with the mantle skirt and the visceral mass. The second cut, separating the anterior and posterior cephalopedal masses, was made immediately anterior to the cavity formed when the visceral mass was cut away from the snail (Fig. 2B). The mantle skirt was then cut away from the visceral mass (Fig. 2C). This method was adapted from Chan *et al*. (2015); but the boundary between the head and foot is not anatomically distinct, so the term ‘cephalopedal mass’ was used, after Hickman (1985). Four snail regions were independently compressed using glass slides, from which a total of 60 glass slides were made. *Angiostrongylus cantonensis* L3 were identified morphologically (Ash, 1970; Bhaibulaya, 1975; Lv *et al*., 2009) and counted manually by microscopy (Fig. 3) (Qvarnstrom *et al*., 2007).

### Larval release in water

Compressed snail tissue from two snails containing L3 (37, 38, 44, 52, 55, 70, 87 days post-infection) used in the larval distribution experiment was transferred off the glass slides into a Petri dish. Snail tissue was submerged in 20 mL of RO water and kept at room temperature (~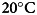). The larval viability experiment was then set at day 0 at this time point. There were a total of 12 Petri dishes established in this study, and each Petri dish contained free-swimming L3 submerging in water over different time courses. L3 emerging from the snail tissues were described in this study as free-swimming L3; these larvae were observed within the Petri dish over time and randomly selected for viability staining using propidium iodide (PI). Other free-swimming L3 in the Petri dishes were used for infecting rats.

### Free-swimming L3 viability using propidium iodide

After release from snail tissue and storage in water at 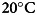, free-swimming L3 progressively became inactive over time, but lack of larval mobility does not always indicate death nor precludes the potential for infectivity (Jarvi *et al*., 2019). Thus, PI was used to circumvent this intrinsic difficulty without interfering with larval infectivity for rats (Jarvi *et al*., 2019), and to investigate their survival in water over time. This stain permeates into cells of dead larvae in which the cell membrane is irrevocably damaged (Zhao *et al*., 2010; Tawakoli *et al*., 2013; Jarvi *et al*., 2019), and binds to nucleic acids in the cell, resulting in red fluorescence staining of the worm when viewed in fluorescent microscopy (Zhou *et al*., 2011).

**Fig. 4.**
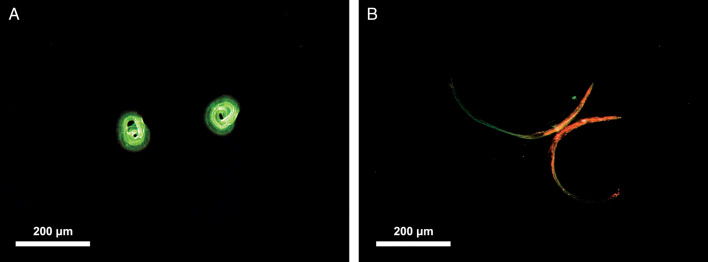
Appearance of *Angiostrongylus cantonensis* free-swimming L3 using propidium iodide staining by fluorescent microscopy. There are two larvae in each picture. (A) Live larvae are coiled with green fluorescence. (B) Dead larvae taking up the PI stain.

The method for PI staining of *A. cantonensis* larvae was performed according to Jarvi *et al*. (2019), with modifications. Briefly, stock aliquots of PI were obtained from Annexin V-FITC Apoptosis Staining/Detection Kit ab14085 (Abcam, Cambridge, UK). A 5% PI solution diluted using RO water was found to be the optimum concentration in preliminary experiments; this is four times the concentration that was used by Jarvi *et al*. (2019). Twenty free-swimming L3 were collected from each Petri dish into a 2 mL Eppendorf tube using a micropipette, to which RO water was added to a total volume of 360 μL. PI solution (40 μL of 5%) was then added. After gentle agitation, tubes were incubated at room temperature in the dark for 30 min. The PI was removed by washing twice in RO water. Larvae were transferred on to a glass slide and examined using fluorescent microscopy at excitation wavelengths of 470 and 555 nm, both at 100% intensity. Live larvae appeared pale green (Fig. 4A), due to a counterstain, while the dead larvae stained a vibrant red colour (Fig. 4B), as described by Kirchhoff and Cypionka (2017). Although some larvae were lost during PI staining, the larval recovery rate of staining procedure was greater than 74% larvae recovered each time.

### Free-swimming L3 infectivity in rats

A total of 16 male Wistar rats (*R. norvegicus*), previously infected with 20 000 *Strongyloides ratti* L3 at least one month prior, were further challenged with 30 free-swimming *A. cantonensis* L3. Although the rats were not cleared of *S. ratti*, all rats received the same dual exposure. The number of surviving *A. cantonensis* adults found in lungs at necropsy had no obvious interference by *S. ratti* infection in Wistar rats (R. Lee, 2020, unpublished observations), corroborating the findings of numerous other studies (Gardner *et al*., 2006; Lv *et al*., 2009; Sakura and Uga, 2010; Viney and Lok, 2015).

Before L3 collection could occur, they needed at least 1 day to migrate away from the snail tissue after dissection, so harvesting of free-swimming living L3 used to challenge rats began 1 day later. Some L3 were inactive larvae (21, 35 and 42 days post-dissection of the snail), so the live–dead status of larvae was determined using PI staining before L3 were selected for infecting rats. Two rats were each infected with 30 free-swimming L3 stored in water for a given time course (1, 7, 14, 21, 28, 35, 42 and 43 days). Each rat was lightly anaesthetised with 5% isoflurane in 100% oxygen. Free-swimming L3 were instilled into the oesophagus using a plastic Pasteur pipette. Fourteen weeks later, rats were euthanised and necropsied. Faeces acquired from the rectum or descending colon (at post-mortem examination) was examined for the presence of L1. Adult worms were retrieved from the pulmonary arteries of lungs using the examination method described by Wallace and Rosen (1965), but without flushing the lungs with water. Gross pathological changes affecting the lungs, such as swollen lobes, discolouration and egg nests (Mackerras and Sandars, 1955; Wun *et al*., 2021*a*), were recorded. The study was conducted under ethics approval from Western Sydney Local Health District Animal Ethics, protocol # 8003.03.18.

### Statistical analysis

**Fig. 5.**
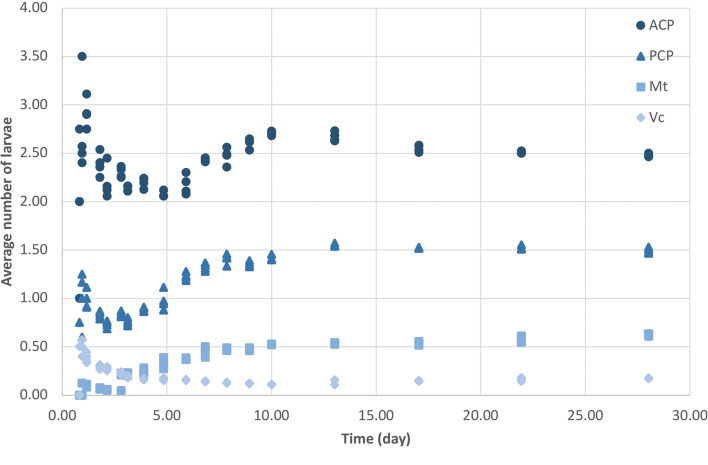
Average *Angiostrongylus cantonensis* larvae detections in four regions of *Bullastra lessoni* snails over 28 days post-infection. ACP, anterior cephalopedal mass; PCP, posterior cephalopedal mass; Mt, mantle skirt; Vc, visceral mass. The *x*-axis is the time of days after infection, while the *y*-axis is the average number of larvae per snail detected in histological sections stained with H&E.

To effectively determine the change of detection rate over time in the larval migration experiment, larval detection data in four snail regions were transformed into cumulative models and averaged by the number of snails. Four measurements acquired from each specimen from the same snail part and time group were evaluated to establish the central tendency and variance for each time point. Temporal trends in the data were modelled using average numbers of larvae, and detection rates, starting from 0.82 days post-infection when multiple larvae were detected (Table 1), were compared statistically by comparing all four trends using a two-factor, repeated-measures analysis of variance (RM ANOVA; Greenhouse–Geisser correction), followed by RM ANOVA and Tukey's *post hoc* test for the pairwise pattern of differences in the number of larvae detected among four snail regions. Due to major alteration in the patterns of average larval detection before and after 10 days post-infection (Fig. 5), 10 days post-infection was determined to be the cut-off point. Subsequent analyses on the average larval numbers before and after 10 days were performed accordingly.

**Table 1.** Total number and percentage of *Angiostrongylus cantonensis* larvae detected in each *Bullastra lessoni* snail part at each time point post-infection (*n* = 96 snails)

Group	Post-infection time (day)	ACP	PCP	Mt	Vc
1	0.00	0	0	0	0
2	0.02	0	0	0	0
3	0.04	0	0	0	0
4	0.08	1 (100.0)	0 (0.0)	0 (0.0)	0 (0.0)
5	0.13	0	0	0	0
6	0.17	1 (100.0)	0 (0.0)	0 (0.0)	0 (0.0)
7	0.82	11 (68.8)	3 (18.8)	0 (0.0)	2 (12.5)
8	0.94	17 (63.0)	7 (25.9)	1 (3.7)	2 (7.4)
9	1.16	5 (83.3)	1 (16.7)	0 (0.0)	0 (0.0)
10	1.80	3 (50.0)	2 (33.3)	0 (0.0)	1 (16.7)
11	2.13	13 (86.7)	2 (13.3)	0 (0.0)	0 (0.0)
12	2.80	5 (35.7)	5 (35.7)	4 (28.6)	0 (0.0)
13	3.13	5 (83.3)	0 (0.0)	1 (16.7)	0 (0.0)
14	3.90	9 (42.9)	9 (42.9)	3 (14.3)	0 (0.0)
15	4.83	6 (26.1)	11 (47.8)	5 (21.7)	1 (4.3)
16	5.92	18 (60.0)	11 (36.7)	1 (3.3)	0 (0.0)
17	6.83	14 (46.7)	9 (30.0)	7 (23.3)	0 (0.0)
18	7.85	17 (68.0)	8 (32.0)	0 (0.0)	0 (0.0)
19	8.94	13 (76.5)	1 (5.9)	3 (17.6)	0 (0.0)
20	10.00	17 (43.6)	17 (43.6)	5 (12.8)	0 (0.0)
21	13.02	2 (18.2)	5 (45.5)	1 (9.1)	3 (27.3)
22	17.05	5 (33.3)	6 (40.0)	4 (26.7)	0 (0.0)
23	21.94	8 (32.0)	7 (28.0)	7 (28.0)	3 (12.0)
24	28.02	7 (70.0)	0 (0.0)	3 (30.0)	0 (0.0)

ACP, anterior cephalopedal mass; PCP, posterior cephalopedal mass; Mt, mantle skirt; Vc, visceral mass.

Meanwhile, in the larval distribution experiment, the temporal trend in the total number of L3 observed in snails dissected in 5, 6, 7, 8, 10 and 12 weeks post-infection was tested using different statistical models (linear, quadratic, logarithmic, growth, and exponential). A *χ*^2^ test was performed to determine if the distribution of L3 among the four snail regions was even. Raw data for the number of L3 found in the various anatomical locations within all 15 snails were evaluated for distribution normality using a Kolmogorov–Smirnov (KS) test, and for variance homogeneity using Bartlett's test. Data were thus transformed using arcsine square root to improve homogeneity of the variance. Transformed data were analysed with a two-factor, general linear model ANOVA with the fixed orthogonal factors DAY, representing the day of infection for a temporally independent design (i.e. different snails were sampled for the different times since infection), and LOCATION, being the part of the snails in which the larvae were detected. Significant differences among the study groups were determined using Tukey's HSD test.

Finally, mortality data, obtained from PI staining, were analysed using *PAST 4.03* (Hammer *et al*., 2001). As some free-swimming L3 might be lost during washing, the recovery rate was calculated as follows:

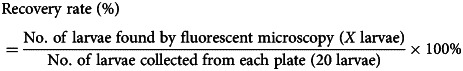


The infectivity of free-swimming L3 was calculated as follows:
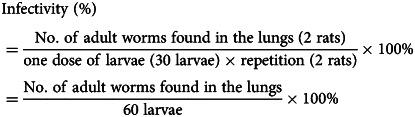


## Results

### Larval migration

A total of 160 snails were left in contact with *A. cantonensis* L1 for 4 hours and washed in RO water, and 96 infected snails were used in this experiment. Larvae were quantified in histological sections within body regions of each of the snail group sacrificed sequentially. The earliest detection of a single larva in the snail tissue was at about 2 hours (Table 1). The total number and percentage of larvae at each designated location within the snail and time point showed most obvious changes within cephalopedal masses and the mantle skirt. In addition, first moult for *Angiostrongylus* L1 happens at around 7–10 days and second moult at 15 days (Mackerras and Sandars, 1955; Bhaibulaya, 1975). The larval stage could not be verified via morphology in histological sections.

The average larval detections in all four snail regions over 28 days post-infection are shown in Fig. 5, and the changes in average larval detections at each snail part and time point over the course of 28 days post-infection were significant (two-factor RM ANOVA: *F* = 10.63, *P* = 0.0000 for the DAY × LOCATION interaction term; Supplementary Table 1). In addition, the average number of larvae detected in each snail part was significantly different (RM ANOVA: *F* = 283.6, *P* = 0.0000; Supplementary Table 2). All pairwise differences of average larval detections in each snail part over the course of 28 days post-infection were significant (*α* = 0.05), except between the mantle skirt and visceral mass.

Since there was an observable alteration in the pattern of average larval detections before and after the first 10 days of infection, we analysed the changes in average larval detections in two separate parts, the first 10 days post-infection and then from 10 to 28 days post-infections, using two-factor RM ANOVA for the DAY × LOCATION interaction term. Over the first 10 days post-infection, the changes were substantial and statistically significant (*F* = 8.153, *P* = 0.0000), but due to the complexity of these changes in larvae numbers, curves of each snail part could not be fitted into any model. The variation in the average larval numbers of all snail regions after 10 days post-infection decreased but was still statistically significant (*F* = 42.02, *P* = 0.0000). The average larval numbers over 10 − 28 days post-infection were fitted into linear model, with the slope of all four linear trendlines close to zero (Supplementary Fig. 1).

### Larval distribution

**Fig. 6.**
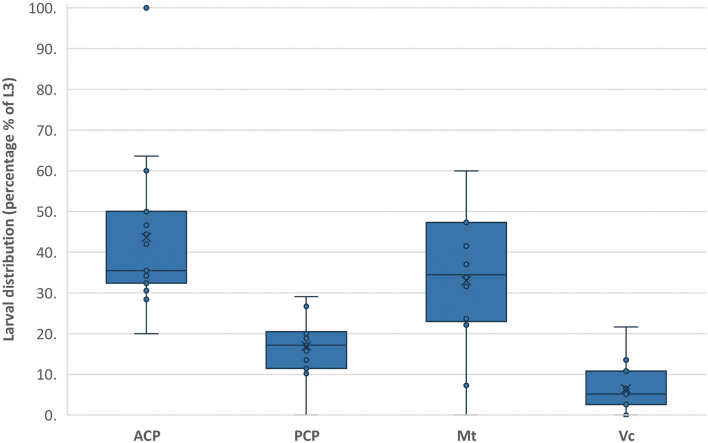
Box–Whisker plot of *Angiostrongylus cantonensis* L3 distribution in *Bullastra lessoni* snails (*n* = 15 snails). ACP, anterior cephalopedal mass; PCP, posterior cephalopedal mass; Mt, mantle skirt; Vc, visceral mass. The box represents the IQR; the line and X within the box represent the median and mean respectively; the ‘whisker’ extends to data points that were 5 − 95% data range; the dot represents a single outlier. The *y*-axis refers to the percentage of larvae present in each anatomical compartment.

Fifteen snails were dissected 5–12 weeks post-infection. The total number of L3 retrieved from each snail ranged from 1 to 431 [mean = 140.8; median = 131; interquartile range (IQR) = 64.5–197.5], and no significant temporal trend was detected in the total number of larvae in each snail (*P* > 0.05 for all models; Supplementary Fig. 2). The primary sites where the larvae were detected were the anterior cephalopedal mass and the mantle skirt [43.6 ± 10.8 and 33.0 ± 8.8%, respectively (mean ± 95% CI)] (Fig. 6). Lower numbers of larvae were also found in the posterior cephalopedal mass (16.9 ± 4.2%) and the visceral mass (6.5 ± 3.3%).

The *χ*^2^ analysis indicated that the distribution of larvae among the four designated snail regions was uneven (*χ*^2^ = 238.8; *P* = 0.0000). The raw data for the proportion of larvae found in the various locations within all 15 snails were normally distributed (KS: *P* > 0.150). As the variances were heterogeneous (Bartlett's test: *P* = 0.022), the data were transformed. The transformed data were normally distributed (KS: *P* > 0.150) and homogeneity of variances improved (*P* = 0.050).

As we used a temporally independent design (i.e. different samples were analysed at each time point), data could be validly analysed with a two-factor ANOVA rather than a repeated measures analysis. This evaluation indicated that there was a significant difference among the proportions of larvae found in the different snail regions (ANOVA: *P* = 0.000), which was not related to the time post-infection in days (*P* = 0.848 for the DAY × LOCATION interaction term; Supplementary Table 3). Different proportions of larval numbers were found between the anterior and the posterior cephalopedal mass (*P* = 0.0016), between the anterior cephalopedal mass and the visceral mass (*P* = 0.0000) and also between the mantle skirt and the visceral mass (*P* = 0.0009). All other pairwise comparisons were not significant (*α* = 0.05).

### Free-swimming L3 viability using propidium iodide

**Fig. 7.**
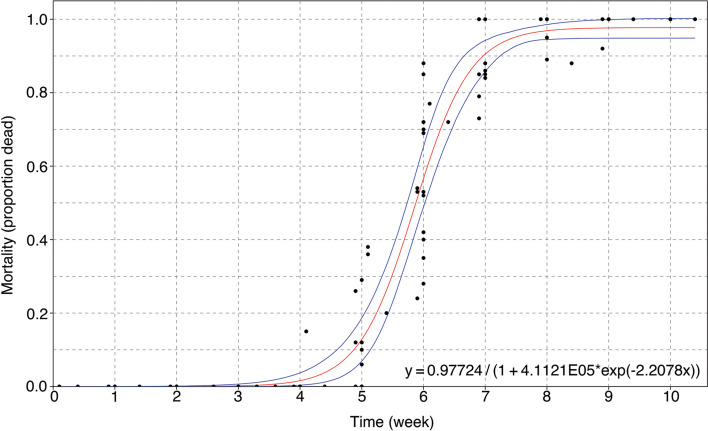
Vital status of *Angiostrongylus cantonensis* free-swimming L3 over time (95% CI are shown). The *x*-axis is the time of weeks after leaving the dead snail hosts, while the *y*-axis is the percentage of free-swimming L3 found dead using PI.

All L3 released from snails survived for the first 4 weeks in RO water (Fig. 7). From that time point, mortality of the free-swimming L3 increased exponentially with time until 8 weeks, when approximately 100% larval mortality was observed. One free-swimming L3 was found alive by week 9, with a total of 112 free-swimming L3 tested, and no free-swimming L3 were found alive by week 10, with a total of 99 L3 tested. The larval mortality was fitted to a logistic model [*y* = 0.97724/(1 + 4.1121 × 10^5^ × *e*^−2.2078*x*^)].

### Free-swimming L3 infectivity in rats

Rats were euthanised and necropsied 14 weeks after being challenged with free-swimming L3 stored in water at approximately 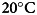. Adult worms were retrieved from the right atrium and pulmonary arteries of infected rats. The number of adult worms harvested from two rats, which were each given a challenge of 30 L3, collected 1 day after release from snail tissue was 38% (23/60; baseline infection). The number of adult worms dissected from rats which were challenged with L3 stored in water for 7 and 14 days were 47% (28/60) and 40% (24/60), respectively. Rats infected with L3 at these time points showed gross pathological lung lesions, and all rats had L1 in their faeces. No adult worms were retrieved from the rats which were infected with L3 after 21 days incubation in water. These rats did not show any lung pathology, nor were L1 detected in wet faecal preparations.

## Discussion

*Angiostrongylus cantonensis* can cause severe neurological infections across a range of vertebrate hosts including humans and birds. However, the biology of this disease demands greater research emphasis (Barratt *et al*., 2016). Important gaps in knowledge remain, including information concerning mechanisms of transmission of *A. cantonensis* L1 and L3 to their host species. The primary aims of this study were to determine when and how L1 enter the snail, the eventual distribution of larvae within the snail, and the potential viability and infectivity of free-swimming L3 released from dissected snails in freshwater.

### Larval migration and distribution

This study showed the distribution of *A. cantonensis* larvae in *B. lessoni*. Larvae migrated to all internal parts of the snail, with the larval distribution numbers fluctuating over time and a significant change in their average numbers over 28 days post-infection. The highest numbers of L3 were in the anterior cephalopedal mass (43.6 ± 10.8%) and the mantle skirt (33.0 ± 8.8%).

*Angiostrongylus cantonensis* L1 are not active swimmers and rely on the snail to move in close proximity of the larvae to initiate infection. Whether L1 enter the snail body by active penetration, ingestion, or a combination of the two remain unknown (Morassutti *et al*., 2014). As such, the current study showed that after exposure of snails to L1, the first larval detections in all four anatomical areas occurred around the same time at 0.82 days (20 hours) post-infection, indicating L1 actively penetrated into the snails and/or by ingestion as the snail feeds.

After primary host invasion, the larval detections in snails fluctuated over time, indicating that migratory activities of the parasitic larvae within snails might exist. This was confirmed by statistical analysis on the average larval numbers over time, showing larvae tended to migrate within snails from the initial site of entry. Our observation concurred with Tesana *et al*. (2008), using *Pila polita* over the course of 3 months, after infecting this snail orally which was different to the method used in our study. Consistently, *A. cantonensis* larval migratory ability was akin to its related species, *A. costaricensis*, with larvae entering the snail both by ingestion and percutaneously and most migrating to the fibromuscular layer of the foot, the circulatory system, and the kidneys (Mendonça *et al*., 1999; Montresor *et al*., 2008).

Early larval migration in the first 10 days post-infection was prominent, while the migratory activities from 10 days onward decreased to a minimum but still reached statistical difference. The time (~10 days post-infection) when this change in larval migratory activities in the mollusc occurred coincided with the larval development from L1 to L3. In the mollusc, first moult for *Angiostrongylus* L1 occurs at around 7–10 days and second moult at 15 days, as determined in *A. mackerrasae* (Bhaibulaya, 1975) and *A. cantonensis* (Mackerras and Sandars, 1955). Larvae do not shed their sheaths after moulting (Mackerras and Sandars, 1955; Lv *et al*., 2009), and the enclosed sheath might hinder the larval migration, which may explain the decreased variation of average larval numbers after 10 days post-infection in this study. However, further studies are required to affirm this correlation.

Nonetheless, apart from initial larval movement, the results also suggested that eventual distribution of *A. cantonensis* L3 in *B. lessoni* snail was attributed to their exposed surface and snail locomotion. Firstly, the shell is a barrier for the snail, protecting its soft vulnerable internal organs from the dangers of the external environment (Hickman, 1985). It is also plausible that the shell provides protection against L1 and lessens the available surface exposure to infective larvae. The significantly lower larval numbers detected in the visceral mass, compared with the exposed anterior and posterior cephalopedal masses at the initial stage of infection, support this suggestion as these organs are sheltered by the shell. The importance of the shell as a barrier to entry of *A. cantonensis* larvae can be seen in the semi-slug (*P. martensi*), a mollusc with a rudimentary fingernail-like shell on the mid-dorsal section (Hollingsworth *et al*., 2013). L3 were chiefly distributed in the midsection and tail (Jarvi *et al*., 2012), regions not covered by the shell. One earlier study, also using *B. lessoni* as the intermediate host, made similar observations to our study (Chan *et al*., 2015). In another study, larvae of two feline lungworm species (*A. abstrusus* and *T. brevior*) were concentrated in the fibromuscular layer of the foot and the mantle skirt of the common garden snail (*Cornu aspersum*) (Giannelli *et al*., 2015), despite their unique inoculation method (Giannelli *et al*., 2014). Others who used different freshwater snails, the ampullariids *Marisa cornuarietis* (Yousif *et al*., 1980) and *P. polita* (Tesana *et al*., 2008), found that *A. cantonensis* larvae were located mainly in the head/foot and mantle skirt, and the mantle and the visceral organs, respectively.

Although the visceral mass is where the least larvae were found, the routes by which larvae could reach this part of the snail are either through the gastrointestinal tract after ingestion or by penetrating and migrating from other regions of the body as Montresor *et al*. (2008) suggested larvae migratory activities were associated with the circulatory system pathway. Larvae of *A. cantonensis* in snails seem to have a tropism for well-perfused anatomical regions, such as the extensive vascular supply and the unique microenvironment of the foot (Giannelli *et al*., 2015). This correlation of larval distribution with snail physiology might explain the reduction in average larval number in the visceral mass over the first 4 days post-infection, suggesting the larvae moved to other snail regions that are enriched with blood supply, resulting in substantially more larvae in the anterior cephalopedal mass and the mantle skirt. Meanwhile, both anterior and posterior cephalopedal masses are not sheltered by the shell; however, significantly more larvae were observed in the former region than the latter probably because of the forward direction of locomotion. The mantle skirt has parts which are located at the anterior of the snail, but the slightly lower larval count compared to the anterior cephalopedal mass might be attributed to partial shielding of the shell.

Other factors which could influence differences in larval distribution among intermediate host species may be associated with varying degrees of susceptibility, such as molluscan host immune responses, food preferences, and the interaction of the biochemical environment of tissues with this parasite (Mackerras and Sandars, 1955; Wallace and Rosen, 1969*a*; Yousif *et al*., 1980; Tesana *et al*., 2008; Chan *et al*., 2015). Overall, previous studies found larval distribution in their molluscan host were similar to our study (Yousif *et al*., 1980; Chan *et al*., 2015; Giannelli *et al*., 2015), and future studies should compare the accumulation of larvae in body sites between various snail types and slugs.

### Free-swimming L3 viability and infectivity

The mortality of free-swimming L3 was found to follow a logistic model, demonstrating 100% viability until week 4, with a precipitous decline in free-swimming L3 viability and virtually 100% mortality found by week 8. Crucially, infectivity of free-swimming L3 for rats persisted for only 2 weeks after release from dissected snails, with an average rate of 25/60 adult worms being retrieved from the pulmonary arteries when rats were challenged with 30 viable L3, which was in agreement with an earlier study demonstrating approximately 40% of infection rate under optimal conditions (Wallace and Rosen, 1969*b*). Infection with adult *A. cantonensis* was also consistent with observation of gross pathological changes in the lung and identification of L1 in infected rat faeces.

A similar result was obtained in two previous studies that showed free-swimming larvae were viable and active 7 days after leaving the snail (Richards and Merritt, 1967) and or when stimulated with acid at 21 days (Howe *et al*., 2019), but neither of these studies assessed the infectivity. Critically, since transmission pathway through drinking contaminated freshwater with free-swimming *A. cantonensis* L3 was considered viable, as analogous to its feline counterparts (*A. abstrusus* and *T. brevior*) (Giannelli *et al*., 2015), it was essential that the mortality and infectivity of free-swimming L3 be determined concurrently.

Free-swimming *A. cantonensis* L3 were found to remain infective for 2 weeks, which is far longer than previous studies, recording 7 days (Richards and Merritt, 1967) and 60 hours (Crook *et al*., 1971). This suggests that larvae are not capable of establishing a patent infection 3 weeks after leaving snail tissue and living in freshwater at 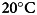; no infection could be established by day 21 under these same conditions. These findings implied that even though free-swimming L3 can remain viable up to 8 weeks after leaving the snail host, their infectivity in rats can only persist for 2 weeks under experimental conditions.

### Study limitations

Molluscan pedal mucus has a protective function (reviewed by Ng *et al*., 2013) and gel-like property (Smith, 2002), and it was possible that a proportion of larvae were trapped in the mucus, thus hindering larval entry into the snail's integument. Variation in mucus production between snail hosts could affect uptake of the larvae in other snail species, so our study findings are limited to *B. lessoni* only.

Whether an increase in larval colonisation of one snail region was at the expense of another region of the snail could not be determined. The changes in transformed larval numbers might also have other confounding factors, such as secondary larval entry into the snail due to inadequate washing of the snail surface. Further studies should be designed to examine larval migration by using L1 labelled with either a radioactive dye or colloidal gold using a monoclonal antibody and tracking actual movement in real time using scintigraphy, positron emission tomography, or functional magnetic resonance imaging, revealing detailed migratory routes of L1, L2, and L3 and the eventual distribution of L3 within its mollusc host.

There were two rationales which contributed to a minor discrepancy between the ranking of larval presence in the migration and distribution experiments. As different visualisation approaches were used in two experiments, the areas of each snail part shown in histological sections were disproportionate to the relative volumes in the three-dimensional viewing of freshly compressed snails because the snail's morphology became distorted when subjected to fixation in formalin. Hence, a longitudinal cut down at the centre of the snail had inherent technical variation as the snail shape was no longer consistent.

During the larval viability experiment, the free-swimming L3 were submerged in RO water at 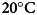. It is unknown whether different storage conditions, such as different temperatures or the removal of snail tissue, could impact on the longevity and infectivity of these larvae.

## Conclusion

*Angiostrongylus cantonensis* is an emerging pathogen. Bridging some of the knowledge gaps to minimise potential transmission of angiostrongyliasis to humans, pets, endangered zoo animals, and other wildlife was the prime objective of this research. We determined that *A. cantonensis* L1 actively penetrated *B. lessoni* snail integument directly and/or subsequently by ingestion, and further migration within the snail of infective larvae was detected over 28 days after initial tissue invasion. Larvae were primarily distributed in the anterior cephalopedal mass and the mantle skirt, followed by the posterior cephalopedal mass and the visceral mass. Lastly, the viability of free-swimming L3 kept in freshwater at 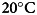 predominantly started to decline after 4 weeks, and no viable larvae were found by 8 weeks. Larval infectivity in rats was only detected up to 2 weeks under these conditions.

## Supplementary material

For supplementary material accompanying this paper visit https://doi.org/10.1017/S0031182022000488. click here to view supplementary material
