# Supplementary material for: Host transmission dynamics of first- and third-stage Angiostrongylus cantonensis larvae in Bullastra lessoni
Source: Parasitology. 2022 Apr 21;149(8):1034–44. doi: 10.1017/S0031182022000488 (PMC10090585; doi:10.1017/S0031182022000488)
Supplement: Supplementary file 1 [file S0031182022000488sup001.docx]

# Supplementary material

**Table 1.** Analysis of variance test (Greenhouse-Geisser) of the significance between time and snail regions in transformed average larval migration data. The data were collected from the detections of *Angiostrongylus cantonensis* larvae in *Bullastra lessoni* histological sections over 28 days post-infection.

| Source | Sum of Squares | DF | Mean Square | F (DFn, DFd) | P-value |
| --- | --- | --- | --- | --- | --- |
| DAY | 8.932 | 51 | 0.1751 | F (51, 204) = 10.63 | 0.0000 |
| LOCATION | 8.987 | 17 | 0.5286 | F (1.578, 18.93) = 32.09 | 0.0000 |
| DAY*LOCATION | 224.2 | 3 | 74.74 | F (3, 12) = 2983 | 0.0000 |
| SNAILS | 0.3007 | 12 | 0.02506 | F (12, 204) = 1.521 | 0.1187 |
| Error | 3.361 | 204 | 0.01647 |  |  |

**Table 2.** Analysis of variance test of the significance among four snail regions in transformed average larval migration data. The data were collected from the detections of *Angiostrongylus cantonensis* larvae in *Bullastra lessoni* histological sections over 28 days post-infection.

| Source | Sum of Squares | DF | Mean Square | F (DFn, DFd) | P-value |
| --- | --- | --- | --- | --- | --- |
| Treatment | 56.05 | 3 | 18.68 | F (3, 68) = 283.6 | 0.0000 |
| Residual | 4.480 | 68 | 0.06588 |  |  |
| Total | 60.53 | 71 |  |  |  |

**Figure 1.** Average *Angiostrongylus cantonensis* larvae detections in four regions of *Bullastra lessoni* snails over 10-28 days post-infection, fitted with linear models, in the larval migration experiment. ACP = anterior cephalopedal mass; PCP = posterior cephalopedal mass; Mt = mantle skirt; Vc = visceral mass. The x-axis is the time of days after infection, while the y-axis is the average number of larvae per snail detected in histological sections stained with H&E.

**Figure 2.** Total *Angiostrongylus cantoensis* L3 detections in each *Bullastra lessoni* snail from 5 to 12 weeks post-infection in the larval distribution experiment.

**Table 3.** Analysis of variance test for arcsine square root transformed larval distribution data. The data were collected from the eventual distribution of *Angiostrongylus cantonensis* L3 in *Bullastra lessoni* fresh tissues at least 5 weeks post-infection.

| Source | DF | Adj MS | F | P-value |
| --- | --- | --- | --- | --- |
| DAY | 5 | 0.00625 | 0.13 | 0.983 |
| LOCATION | 3 | 0.65158 | 14.00 | 0.000 |
| DAY*LOCATION | 15 | 0.02836 | 0.61 | 0.848 |
| ERROR | 36 | 0.04654 |  |  |
